# The Potential Pathway Among Self-Focused Attention, Rumination, Perceived Stress and Female Psychosomatic Symptoms

**DOI:** 10.3390/bs16081266

**Published:** 2026-07-23

**Authors:** Moya Xu, Shulin Chen

**Affiliations:** Department of Psychology and Behavioral Sciences, Zhejiang University, Hangzhou 310058, China; 12339007@zju.edu.cn

**Keywords:** female, psychosomatic symptoms, self-focused attention, rumination, perceived stress

## Abstract

Psychosomatic symptoms are common among females and involve complex psychological and cognitive processes. This study examined the relationships among self-focused attention (SFA), rumination, perceived stress, and psychosomatic symptoms. A cross-sectional survey was conducted in China among 839 women (mean age = 26.50 ± 6.08 years). Standardized questionnaires assessed SFA, rumination, perceived stress, anxiety symptoms, and somatic symptoms. Correlation, hierarchical regression, and structural equation modeling (SEM) analyses were performed. Psychosomatic symptoms were positively correlated with perceived stress, rumination, and SFA. SFA was positively correlated with rumination but not with perceived stress. SEM indicated a full mediation pattern in which rumination and perceived stress mediated the relationship between SFA and psychosomatic symptoms. The indirect path through rumination alone was not significant, whereas the indirect path through perceived stress alone and the chained indirect path through rumination and perceived stress were significant. The final SEM fit indices were (χ^2^ [70] = 441.460, χ^2^/df = 6.307, CFI = 0.965, IFI = 0.965, TLI = 0.954, RMSEA = 0.080 [0.073–0.087], SRMR = 0.079, RMR = 0.037). The findings suggest that SFA may be associated with psychosomatic symptoms through different pathways. Given the online convenience sampling and the relatively limited age distribution, the findings should be generalized cautiously to broader female populations.

## 1. Introduction

Psychosomatic disorders are characterized by symptom clusters that reflect interactions between psychological and physical dysfunction, as well as the co-occurrence of syndromes (Ulyukin et al., 2021). They are generally defined as physical illnesses or symptom presentations in which psychological distress and stress-related processes significantly affect somatic functioning (Nisar & Srivastava, 2017). Such conditions, including somatization-related symptoms that are triggered or exacerbated by psychological factors, chronic pain, and functional gastrointestinal problems, have been reported to be more prevalent in females (Kajantie & Phillips, 2006). Higher levels of anxiety and depression have also been consistently observed in females than in males (Farhane-Medina et al., 2022; Ussher, 2010). In addition, female-specific hormonal fluctuations, such as those occurring during the menstrual cycle, pregnancy, the postpartum period, and menopause, have been shown to exert substantial effects on mental health. From a sociological perspective, females may also be more vulnerable to multiple stressors, including the unequal distribution of traditional family roles and caregiving responsibilities, as well as social pressures related to appearance and achievement (Cherepanov et al., 2010; Divya & Devi, 2024). However, existing diagnostic labels offer limited explanatory power, and the mechanisms underlying these symptoms remain controversial. Psychosomatic connections are therefore crucial for understanding the complex interactions between psychological and physical states, particularly in the context of health and disease. This bidirectional relationship suggests that psychological states may affect physical health, whereas physical conditions may also exert significant effects on psychological functioning.

### 1.1. Potential Mediating Effect of Rumination Between SFA and Psychosomatic Symptoms

Psychosomatic symptoms, including sensations, arousal, physical symptoms, and emotions, constitute a self-perceived experience (Kurlansik & Maffei, 2016). Coping strategies such as avoidance, behavioral disengagement, and substance use are often directed toward reducing negative experiences rather than addressing their underlying sources (Ottenbreit & Dobson, 2004; Penley et al., 2002). From this perspective, attentional processes may be especially relevant to psychosomatic symptoms. Self-focused attention (SFA) refers to the tendency to direct attention inward, away from the external environment, and toward internal states and self-referential information (Ingram, 1990). According to conventional views, high SFA is considered maladaptive and is associated with various mental disorders. Heightened SFA has frequently been observed in a range of psychopathologies, including depression, anxiety, and schizophrenia, and has often been linked to maladaptive cognitive processes (Woodruff-Borden et al., 2001). Since SFA may increase the likelihood that internal information enters conscious awareness, it may also increase sensitivity to bodily experiences and somatic discomfort. In this sense, SFA may be associated with a greater frequency of somatic symptoms and with more intense perceptions of bodily activity. SFA has also been regarded as an indicator of negative cognitive patterns that are themselves related to depression and anxiety, particularly under stress (Oliver et al., 1995). However, recent research has indicated that SFA is neutral in itself, and its effects depend on the underlying cognitive processing mechanisms (Trapnell & Campbell, 1999).

Previous studies have shown that rumination is associated with SFA, such as one study primarily describing this in the context of social anxiety disorder (Hofmann, 2007). Rumination refers to a repetitive tendency to focus on distress symptoms and on their possible causes and consequences without engaging in active problem solving (Nolen-Hoeksema et al., 2008). Post-event rumination frequently occurs following unsuccessful or ambiguously effective social interactions, particularly when individuals perceive high social costs and develop negative self-perceptions due to anticipated catastrophic outcomes (Hofmann, 2007). Research in non-clinical university samples has further shown that trait self-focus predicts subsequent increases in rumination. In addition, several studies have suggested that SFA is associated with greater rumination among adolescents (Gaydukevych & Kocovski, 2012; Holzman & Valentiner, 2016). In a female-dominated sample, SFA was found to be more strongly related to negative affect, with rumination identified as one of the mediating variables, and the effects associated with rumination were stronger than those linked to non-ruminative self-focus (Mor & Winquist, 2002).

Existing studies have provided strong evidence that rumination has a significant impact on both physical and psychological symptoms. In the field of physical health, rumination appears to be associated with symptom exacerbation and poorer clinical outcomes, a relationship that is particularly evident in the context of pain (Sansone & Sansone, 2012). Furthermore, research has confirmed that rumination leads to physical discomfort, including delayed cortisol responses and prolonged cardiovascular reactions (Busch et al., 2017; Zoccola et al., 2008; Zoccola & Dickerson, 2012). In the context of mental health, the association between rumination and symptoms is also highly significant. A study involving two longitudinal studies found that rumination can serve as a cross-diagnostic factor for depression and anxiety (McLaughlin & Nolen-Hoeksema, 2011). A meta-analysis of 179 correlational studies and 37 clinical group comparison studies found that rumination has a significant impact on both anxiety and depression (Olatunji et al., 2013). In light of the above evidence, this study proposes the following hypothesis:

**H1.**  *Rumination mediates the relationship between SFA and psychosomatic symptoms.*

### 1.2. Potential Mediating Effect of Perceived Stress Between SFA and Psychosomatic Symptoms

Stress perception refers to an individual’s subjective experience that the demands of the living environment exceed the resources available to cope with them (Lazarus & Folkman, 1984). Perceived stress is defined as the psychological response generated through cognitive appraisal processes when individuals encounter threatening environmental stimuli (Lazarus, 1984). Numerous studies have confirmed that high levels of perceived stress are a direct risk factor for physical and mental health symptoms (Cohen et al., 2007), which can be explained to some extent by biological processes. Perceived stress has been identified as an important biopsychosocial factor associated with female psychosomatic health (Hange et al., 2013). Changes in health status are generally understood to arise from interactions among the central nervous, endocrine, and immune systems (Glaser & Kiecolt-Glaser, 2005). At the neuroendocrine level, persistent stress has been shown to chronically activate the hypothalamic–pituitary–adrenal (HPA) axis (Cherian et al., 2019). Adrenocorticotropic hormone (ACTH) is then released by the pituitary gland, promoting glucocorticoid synthesis and resulting in sustained elevations in cortisol. These changes may affect multiple physiological systems and may help explain why women experiencing high stress are more susceptible to metabolic syndrome and allergic disorders (Lightman et al., 2020). Stress has also been shown to promote the release of pro-inflammatory cytokines within the immune system (Qing et al., 2020). In addition, cerebral sensitization may be amplified through vagal afferent signaling, thereby lowering the threshold for perceiving physiological signals such as pain (Ravi et al., 2021). Immune responses may also be altered by stress, aggravating autoimmune conditions and delaying healing processes (Alotiby, 2024; Nunez et al., 2025). Beyond its effects on immune functioning, stress has been associated with pain sensitivity, particularly in chronic conditions such as chronic fatigue syndrome and fibromyalgia (Gupta & Silman, 2004; Wyns et al., 2023). Perceived stress has likewise been positively associated with a range of psychosomatic symptoms, including peptic ulcers, migraines, and musculoskeletal pain (Deding et al., 2016; Moon et al., 2017; Østerås et al., 2015). Thus, the association between perceived stress and psychosomatic symptoms appears to be both broad and pervasive across populations. Among university students, a significant positive association between perceived stress and psychosomatic symptoms has been reported, with this pattern appearing to be especially pronounced in females (Heinen et al., 2017; Teixeira et al., 2022). Similar findings have also been reported among nursing populations (Jaradat et al., 2016). Comparable evidence has been observed across different stages of women’s lives, including nulliparous, pregnant, peripartum, and postpartum populations, particularly with respect to depression and anxiety (Bann et al., 2017; Gollenberg et al., 2010; Razurel et al., 2017; Staneva et al., 2015).

Previous studies have reported associations between SFA and perceived stress, with the direction of this relationship remaining inconsistent. Among athletes, learning skills in a highly self-focused environment leads to excessive attention to the motor process, thereby amplifying the negative impact of self-focus on performance under stress (Liao & Masters, 2002). In individuals with social anxiety, SFA is thought to reflect attempts to control or alter internal experiences, suggesting a negative correlation with perceived stress (Glick & Orsillo, 2011). Similar negative relationships have been observed in studies of chronic workplace stress, as self-focus may buffer the impact of acute life events (Frone & McFarlin, 1989). Based on these insights, the following hypothesis is proposed:

**H2.**  *Perceived stress mediates the relationship between SFA and psychosomatic symptoms.*

### 1.3. Rumination and Perceived Stress

A substantial body of research has suggested that rumination and perceived stress are relevant to a range of psychological disorders, and that these variables may interact with each other, although findings are inconsistent. For instance, rumination influences multiple vital psychological processes in individuals with fibromyalgia and is a significant contributor to their stress levels (Malin & Littlejohn, 2015). In premenstrual dysphoric disorder (PMDD), premenstrual symptoms, perceived stress, and daily rumination may mutually reinforce each other, forming a vicious cycle that maintains and exacerbates premenstrual symptoms over the long term (Nayman et al., 2023). Though rumination has been identified as a significant predictor of PMDD (Kaluve & Graham, 2025), perceived stress is not always predictive (Klatzkin et al., 2006; Lee & Im, 2016; Liu et al., 2024; Roomaney & Lourens, 2020). In depression, stress significantly moderates the relationship between rumination and depressive symptoms, yet gender influences this moderation pattern. A study has revealed the moderating effects of stress and rumination on depressive symptoms. The association between rumination and depressive symptoms in females appears to be influenced by stress levels, whereas in males, the link between rumination and depression disappears when stress levels are low. In another model, rumination moderates the relationship between stress and depressive symptoms, further highlighting the existence of gender differences. For females, rumination levels influence the extent to which stress affects depressive symptoms; for males, rumination simultaneously affects both stress and depressive symptoms regardless of its level (Mezo & Baker, 2012). On the other hand, rumination has a mediating role in the association between perceived stress and depressive symptoms, which has been validated among college students (Du et al., 2018; Feng et al., 2024). Additionally, individuals prone to rumination exhibit higher levels of post-traumatic stress symptoms (Xu et al., 2022; Zhang & Ye, 2022).

Research on stress, coping, and disease has often paid insufficient attention to persistent cognitive phenomena such as worry, rumination, and other similar symptoms. Most stress research has focused on stimulus characteristics or individuals’ perceptions of those stimuli, with comparatively less attention given to enduring cognitive processes that may help account for associations with health outcomes (Brosschot et al., 2006). The perseverative cognition hypothesis proposes that stress may exert chronic effects on both physical and mental health. These effects have been linked not only to depression and anxiety, but also to physiological systems such as the cardiovascular, autonomic nervous, and endocrine systems (Brosschot et al., 2006). Rumination, as a prototypical form of perseverative cognition, is characterized by repetitive, intrusive, negative cognitions (Nolen-Hoeksema et al., 2008; Papageorgion & Siegle, 2003). It may be understood as an extendable cognitive process and may function as a cognitive extension of SFA (Jacobs et al., 2016; Williams & Moulds, 2007). Experimental evidence has shown that induced rumination has adverse effects on mood and increases physiological arousal, whereas attentional diversion or conscious self-monitoring may buffer these effects (Nolen-Hoeksema et al., 2008). A growing body of research further indicates that rumination about stressful events is positively correlated with individual stress levels and psychosomatic symptoms (Denovan et al., 2019; Larionow et al., 2022; Marcus et al., 2008; Miers et al., 2007; Watkins, 2004). These findings highlight rumination as a key cognitive mechanism through which stress-related processes may be prolonged and intensified, thereby increasing vulnerability to psychosomatic outcomes. In light of the above findings, this study proposes the following research hypothesis:

**H3.**  *Rumination and perceived stress are sequential mediators in the relationship between SFA and psychosomatic symptoms.*

### 1.4. The Present Study

In the current research context, there remains a gap in studies examining the relationship between female SFA, rumination, perceived stress, and psychosomatic symptoms. To address these gaps, this study proposes a structural equation model to examine the associations among these four key variables and to explore whether the observed pattern of relationships is consistent with a chained mediation framework. Through this model, we aim to provide a theoretical perspective and empirical evidence for understanding the interplay between females’ psychological and physical health. In this study, psychosomatic symptoms are defined as a broader symptom burden that includes both manifestations of anxiety-related symptoms and somatic symptom concerns. This operational definition is based on the evidence that physical symptoms often co-occur with anxiety, rather than appearing as entirely independent phenomena. For example, somatization has traditionally been defined as the tendency to experience and express psychological distress as somatic symptoms, reflecting the close link between psychological distress and physical discomfort (Lipowski, 1987). Recent reviews also indicate that somatic symptom disorders are highly comorbid with anxiety and depressive disorders, and that anxiety is typically accompanied by both psychological and somatic manifestations (Löwe et al., 2022; Mallorquí-Bagué et al., 2016; Nabi et al., 2010). Therefore, although anxiety symptoms and somatic symptoms belong to distinct categories, when research focuses on the interaction between cognitive-emotional distress and the experience of somatic symptoms, the two may collectively reflect a comprehensive psychosomatic symptom burden. The present study focuses specifically on rumination and perceived stress as key mediating factors. From the concept of rumination, which refers to the tendency to repeatedly focus on distressing symptoms and their potential causes and consequences (Nolen-Hoeksema, 1991), it can be inferred that rumination may serve as a key link between SFA and an individual’s experience of distress, thereby being associated with the emergence of psychosomatic symptoms. Furthermore, perceived stress, defined as an individual’s subjective experience of external stressors, has been demonstrated to have a strong correlation with psychosomatic symptoms (Kadzikowska-Wrzosek, 2012). This suggests that perceived stress may be relevant to psychosomatic symptoms and may be involved in the associations examined in the present study.

Given the substantial evidence of gender differences in internalizing symptoms and cognitive coping styles, focusing on female samples is particularly meaningful. Previous research has consistently shown that females exhibit higher prevalence rates of internalizing symptoms, such as somatic symptoms, depression and anxiety. Research demonstrates that, regardless of the exclusion of gynecological and reproductive symptoms, the exclusion of all bodily symptoms, or the focus solely on medically unexplained symptoms, females consistently report more intense, numerous, and frequent bodily symptoms than males (Barsky et al., 2001). Data indicate that females are twice as likely as males to experience depression (Kessler, 2006). These symptoms are closely associated with maladaptive cognitive processes (Altemus et al., 2014). As demonstrated by Johnson and Whisman’s study (Johnson & Whisman, 2013), females exhibit a stronger tendency toward rumination and are more susceptible to stress-related disorders. Furthermore, females are more likely to employ ruminative coping strategies when dealing with stress, and such strategies have been identified as key vulnerability factors associated with stress-related psychological and physiological health problems (Nolen-Hoeksema et al., 2008; Nolen-Hoeksema & Jackson, 2001). Thus, the relevance of focusing on women lies not only in the higher prevalence of psychosomatic and internalizing symptoms, but also in the cognitive and stress-related processes that may contribute to these symptoms. These characteristics suggest that females may be particularly sensitive to the pattern of associations among the four variables examined in the present study. Accordingly, the present study examined the proposed chained pathway within a female sample, in order to better understand the psychological processes associated with female psychosomatic symptom burden.

Although traditional biomedical models emphasize physiological factors such as hormonal fluctuations, they fail to explain why women with similar physiological characteristics exhibit significant differences in the severity of psychosomatic symptoms. Also, these models offer limited guidance for subsequent psychological interventions. Therefore, examining psychosomatic symptoms from a psychological perspective is of great significance. Existing evidence suggests that psychological and cognitive factors may exacerbate physiological vulnerability through neuroendocrine pathways (Bhattacharya et al., 2024). Identifying specific cognitive factors and characterizing their role in psychosomatic symptoms can help transform from a broad description of symptoms toward a more clearly defined and testable model. Accordingly, this study examined the relationships among SFA, rumination, perceived stress, and psychosomatic symptoms in females, aiming to provide a foundation for developing more comprehensive female health support strategies in the future. As participants were recruited online through convenience sampling and the sample was relatively young on average with a relatively limited age distribution, the present study focused on testing this model within a specific online adult female sample.

**Figure 1:**
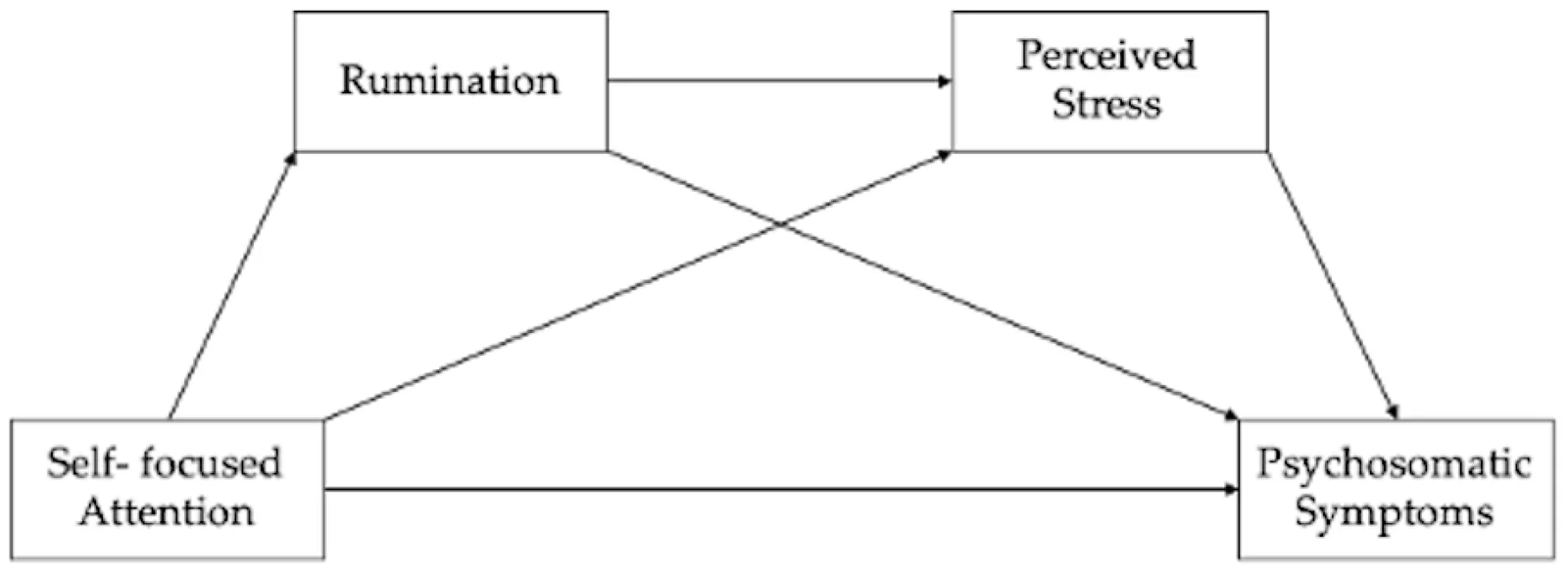
Hypothesis Model.

Based on the above and related hypotheses, a visual model is presented in Figure 1.

## 2. Materials and Methods

### 2.1. Participants

Participants were recruited online via social media platforms (primarily WeChat). Eligibility criteria included being female and aged between 18 and 59 years. A total of 928 individuals participated in the study. After screening for missing or invalid data, 839 valid responses were retained for analysis. All participants provided informed consent and the study adhered to ethical guidelines approved by the institutional ethics committee.

### 2.2. Materials

#### 2.2.1. Self-Focused Attention

Self-focused attention was assessed using the Chinese version of the Self-Focused Attention Scale (SFAS), originally developed by Kiropoulos and Klimidis (2006) and revised by Xiao (2010). The scale contains 17 items across four subscales: Public Body Consciousness, Public Self-Consciousness, Private Self-Consciousness, and Private Body Consciousness. Items are rated on a 5-point Likert scale (1 = “not at all true” to 5 = “completely true”). Higher scores indicate greater self-focused attention. In the present study, the SFAS showed good internal consistency (Cronbach’s α = 0.822).

#### 2.2.2. Rumination

Rumination was measured using the Chinese version of the Ruminative Response Scale (RRS-CV), revised by Han and Yang (2009). The RRS-CV includes 22 items covering three dimensions: compulsive thinking, reflective pondering, and symptomatic rumination. Items are rated on a 4-point Likert scale. Higher scores reflect greater ruminative tendencies. The scale demonstrated excellent reliability in this study (Cronbach’s α = 0.956).

#### 2.2.3. Perceived Stress

Perceived stress was assessed using the 10-item Chinese version of the Perceived Stress Scale (PSS-10), originally developed by Cohen et al. (1983) and revised by Chen et al. (2021). The PSS-10 measures two subdimensions: helplessness *and* self-efficacy beliefs. Items are scored on a 5-point Likert scale, with items 4, 5, 7, and 8 reverse-scored. Higher scores indicate greater perceived stress. The Cronbach’s alpha in this study was 0.838.

#### 2.2.4. Psychosomatic Symptoms

Psychosomatic symptoms were modeled as a latent variable inferred from two scales: The Generalized Anxiety Disorder-7 (GAD-7), which measures the frequency of anxiety symptoms (Spitzer et al., 2006). Each of the 7 items is rated on a 4-point Likert scale ranging from 0 (“not at all”) to 3 (“nearly every day”). Internal consistency in this sample was excellent (Cronbach’s α = 0.925). The second indicator was the Patient Health Questionnaire-15 (PHQ-15), which evaluates the severity of somatic symptoms (Kroenke et al., 2002). Each item is rated similarly on a 0 to 3 scale. The PHQ-15 demonstrated good reliability in this study (Cronbach’s α = 0.865). These two instruments were used as observed variables loading onto a single latent construct of psychosomatic symptoms in the structural equation model.

In the present study, psychosomatic symptoms were conceptualized as a broader symptom burden involving anxiety symptoms and somatic symptoms. Although they are distinguishable domains, they frequently co-occur and jointly reflect the interaction between emotional distress and physical symptom experience. Therefore, the GAD-7 and PHQ-15 were modeled as related indicators of a broader psychosomatic symptom construct.

This approach is consistent with structural equation modeling practices in which a broader latent construct is inferred from multiple related but distinguishable indicators (P. Kline, 2015). To provide additional empirical support for this operationalization, an exploratory factor analysis (EFA) was conducted on the 22 items of the GAD-7 and PHQ-15. In addition, a supplementary parcel-level confirmatory factor analysis (CFA) was conducted to further examine whether anxiety-related symptom indicators from the GAD-7 and somatic symptom indicators from the PHQ-15 could be represented by a common psychosomatic symptom burden construct. The latent structure allows for the simultaneous estimation of shared variance across anxiety-related symptoms and somatic symptomatology, providing a more robust and theoretically coherent representation of psychosomatic symptom burden (Brown, 2015).

Item-level CFAs based on the original scale structures were conducted to provide additional information on the measurement structures of the multidimensional scales, and the results are reported in Supplementary Table S1.

### 2.3. Procedure

Data collection was conducted by using online questionnaire link which was distributed through various social media platforms, including WeChat and campus forums. Participants accessed the survey voluntarily, and only those who provided informed consent could proceed. The survey was self-administered and required approximately 10 min to complete. Participants who completed the survey received a 5RMB reward. To ensure data quality, attention-check items were included.

### 2.4. Data Analyses

All statistical analyses were conducted using IBM SPSS 29.0 and AMOS 30.0. Statistical significance was set at *p* < 0.05 for all inferential tests. Prior to main analyses, Kolmogorov–Smirnov tests were conducted to assess the normality of each continuous variable. Absolute values of skewness and kurtosis below 2 were used as the criterion for acceptable normality (R. B. Kline, 2023). Descriptive statistics were used to summarize participants’ demographic characteristics. Pearson correlation coefficients were computed to examine correlations among key psychological variables. Although SEM was the primary analytical technique used to test the hypothesized model and mediation pathways, preliminary linear regression analyses were also conducted to explore associations between key independent and dependent variables, as an initial step before model specification. To further clarify the inconsistent mediation pattern observed in the SEM, supplementary PROCESS mediation analyses were conducted in SPSS. Rumination and perceived stress were tested separately as single mediators between SFA and psychosomatic symptoms using PROCESS Model 4 with 5000 bootstrap samples. These supplementary analyses were used to aid interpretation of the SEM results.

SEM was employed to examine the hypothesized mediation model. In the model, SFA was treated as an independent variable, rumination and perceived stress were treated as sequential mediators, and psychosomatic symptoms were treated as the latent dependent variable indicated by GAD-7 and PHQ-15 total scores. To reduce the number of estimated parameters and improve model stability, item parceling was applied in the SEM analysis. Following established SEM recommendations (Little et al., 2002; Matsunaga, 2008), items within each scale were assigned to 2–4 parcels. Specifically, the SFAS and RRS-CV items were assigned to four parcels respectively, the PSS-10 items were assigned to three parcels, and the psychosomatic symptom indicators were assigned to three parcels. The mean score of each parcel was used as a manifest indicator in the model. The parcel composition and item assignment matrix are presented in Supplementary Table S2. Path coefficients were estimated using the maximum likelihood method. Model fit was evaluated using multiple indices with acceptable criteria: χ^2^/df < 5; RMSEA < 0.08; SRMR < 0.08; RMR < 0.08; CFI > 0.90; TLI > 0.90; NFI > 0.90; IFI > 0.90 (Hu & Bentler, 1999). Indirect effects were tested using bootstrapping with 5000 resamples and bias-corrected 95% confidence intervals.

## 3. Results

### 3.1. Participant Characteristics

The sample consisted of 839 individuals. The participants ranged in age from 18 to 59 years (M = 26.50, SD = 6.08). Among them, 67.2% were single, 32.2% were married, and 0.6% were divorced or widowed. Regarding occupational status, the largest group consisted of students (n = 218, 26.0%). This was followed by freelance workers (n = 57, 6.8%) and administrative staff (n = 57, 6.8%). Other commonly reported occupations included product/operations staff (n = 50, 6.0%), teachers (n = 44, 5.2%), and self-employed individuals (n = 43, 5.1%). Remaining occupations such as marketing personnel, technical professionals, and service workers, each accounted for less than 5% of the total sample and were grouped under “Other” in subsequent analyses. Detailed demographic characteristics are presented in Table 1.

### 3.2. Descriptive Statistics and Correlation Analysis

Table 2 shows the correlations among the variables being studied to determine which variables to include in the path model. All variables showed satisfactory internal consistency. Psychosomatic symptoms were strongly and positively correlated with both perceived stress (r = 0.696, *p* < 0.01) and rumination (r = 0.759, *p* < 0.01), and weakly correlated with SFA (r = 0.132, *p* < 0.01). Rumination was positively correlated with both SFA (r = 0.265, *p* < 0.01) and perceived stress (r = 0.706, *p* < 0.01). Critically, there was no significant correlation between SFA and perceived stress (r = 0.004, *p* > 0.05), which did not support a direct association between these two variables in the present sample. Overall, the correlation pattern was consistent with the possibility that the association between SFA and psychosomatic symptoms may involve rumination and perceived stress.

As the present study employed a cross-sectional design based on self-report questionnaires, common method bias (CMB) was assessed to evaluate the potential influence of shared measurement methods. Two complementary procedures were conducted. First, Harman’s single-factor test was performed using exploratory factor analysis (EFA), with all measurement items entered simultaneously. The analysis yielded 11 factors with eigenvalues greater than 1 (KMO = 0.970; Bartlett’s test of sphericity = 32159.363, df = 2485, *p* < 0.001), accounting for 58.3% of the total variance. The first factor explained 30.8% of the variability, which was below the recommended threshold of 40%, suggesting that no single factor accounted for the majority of the covariance among the measures (Podsakoff et al., 2012; Zhou & Long, 2004). Second, a single-factor confirmatory factor analysis (CFA) was conducted. The single-factor model demonstrated poor fit (χ^2^ = 73352.897, df = 2485, CFI = 0.788, RMSEA = 0.086, RMR = 0.093, TLI = 0.782), indicating that the observed covariance could not be adequately explained by a single latent factor. Taken together, these findings suggest that common method bias was unlikely to have substantially influenced the results of the present study.

**Table 2:** Pearson correlation coefficients between variables (n = 839).

Variables	Min-Max	Mean (SD)	SK	KU	r		
					2	3	4
1. Psychosomatic Symptoms	0.00–2.50	0.86 (0.54)	0.49	−0.65	0.696 **	0.132 **	0.759 **
2. Perceived Stress	0.00–4.00	1.73 (0.70)	−0.06	−0.12		0.004	0.706 **
3. Self-focused Attention	1.24–5.00	3.73 (0.52)	−0.57	1.10			0.265 **
4. Rumination	1.00–4.00	2.33 (0.67)	0.04	−0.71			

Note. SD Standard Deviation, SK Skewness, KU Kurtosis, r = Pearson’s correlation coefficient, ** *p* < 0.01.

**Figure 2:**
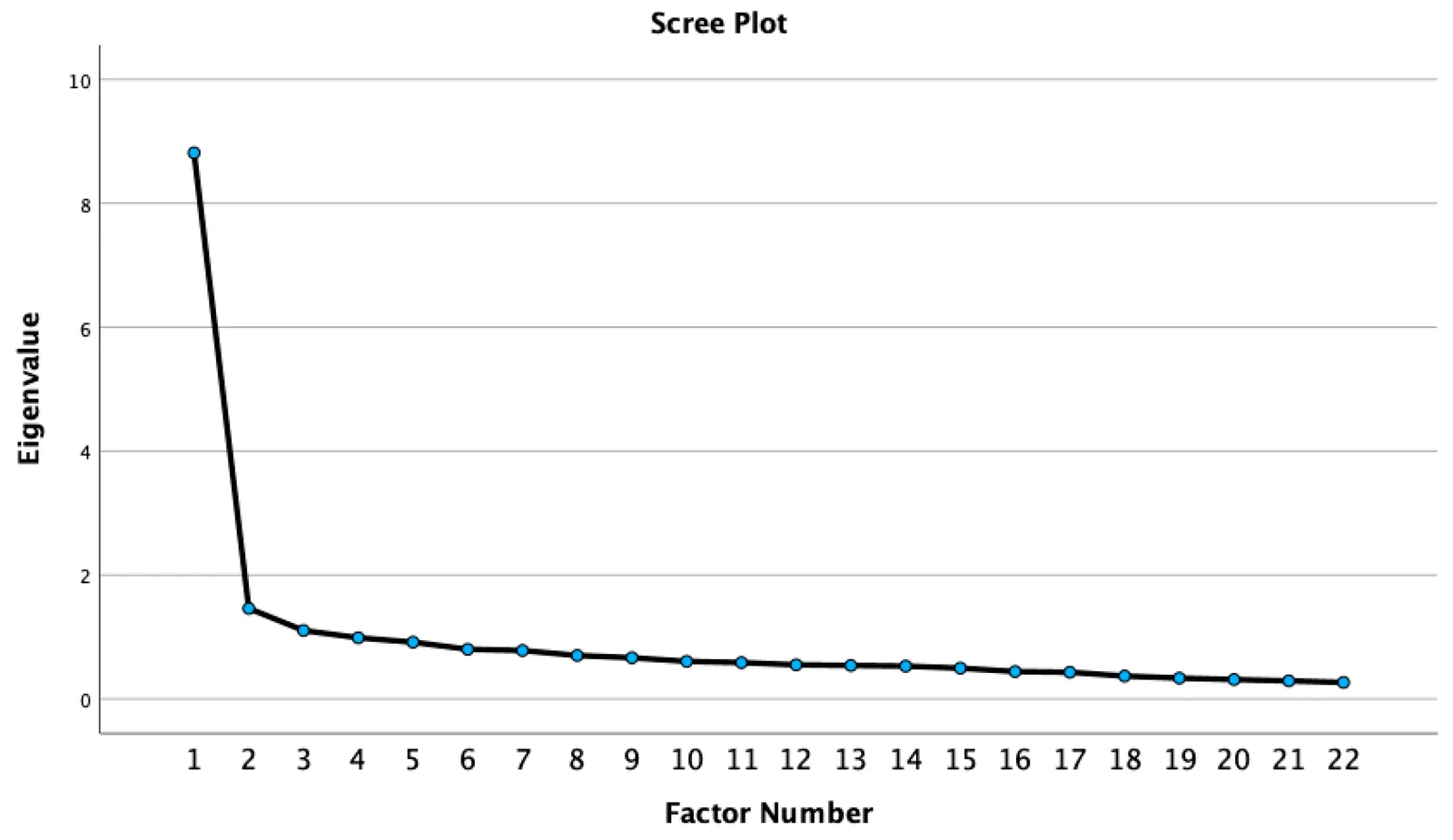
Scree plot of eigenvalues for the exploratory factor analysis of GAD-7 and PHQ-15 items.

To further examine the structural coherence of the psychosomatic symptoms construct, an additional EFA was conducted on the 22 items of the GAD-7 and PHQ-15. The results indicated excellent sampling adequacy (KMO = 0.960) and a significant Bartlett’s test of sphericity (χ^2^ = 8053.102, df = 231, *p* < 0.001). The scree plot is presented in Figure 2 and suggested a dominant first factor, and all items loaded positively on this factor. Detailed item descriptions and factor loadings are presented in Supplementary Material Table S3. A supplementary parcel-level CFA further supported this measurement structure, χ^2^ (8) = 12.114, χ^2^/df = 1.514, CFI = 0.999, IFI = 0.999, TLI = 0.959, RMSEA = 0.025, 90% CI [0.000, 0.051], SRMR = 0.010, and RMR = 0.003.

### 3.3. Hierarchical Regression Analysis

To preliminarily examine the predictive effects of self-focused attention (SFA) and the potential mediators, hierarchical regression analyses were conducted, as shown in Table 3. In Step 1, entering SFA alone yielded a small but significant model, R^2^ = 0.017, F (1, 837) = 14.862, *p* < 0.001. SFA positively predicted symptoms (β = 0.138, *p* < 0.001).

In Step 2, adding rumination substantially improved the model, ΔR^2^ = 0.564 (final R^2^ = 0.582), F (1, 836) = 581.157, *p* < 0.001. Rumination was a strong positive predictor (β = 0.632, *p* < 0.001), and the coefficient for SFA became negative and smaller in magnitude (β = −0.078, *p* = 0.001), suggesting possible mediation via rumination. Notably, the change in the direction of the SFA coefficient may indicate a suppression effect, whereby rumination accounts for variance in SFA that is more strongly associated with psychosomatic symptoms.

In Step 3, entering perceived stress produced an additional increment in explained variance, ΔR^2^ = 0.046 (final R^2^ = 0.628), F (1, 835) = 469.415, *p* < 0.001. In the final model, perceived stress (β = 0.244, *p* < 0.001) and rumination (β = 0.438, *p* < 0.001) were significant positive predictors, whereas SFA was no longer significant (β = −0.013, *p* = 0.591). This pattern was consistent with an indirect association between SFA and psychosomatic symptoms through rumination and perceived stress. Taken together, these regression results were in line with the possibility that the association between SFA and psychosomatic symptoms may be better accounted for by rumination and perceived stress than by a direct effect of SFA alone. Collinearity diagnostics indicated that all variance inflation factor (VIF) values were below 5, suggesting that multicollinearity was not a concern (Hair et al., 2019).

**Table 3:** Hierarchical Regression Predicting Psychosomatic Symptoms (n = 839).

Step	Predictor	β (*p*)	R^2^	ΔR^2^	F (df)	*p*	VIF
1	SFA	0.138 (<0.001)	0.017	0.017	14.862 (1, 837)	<0.001	1.000
2	SFA	−0.078 (0.001)					1.076
	Rumination	0.632 (<0.001)	0.582	0.564	581.157 (1, 836)	<0.001	1.076
3	SFA	−0.013 (0.591)					1.160
	Rumination	0.438 (<0.001)					2.312
	Perceived Stress	0.244 (<0.001)	0.628	0.046	469.415 (1, 835)	<0.001	2.150

Note. SFA Self-focused attention, VIF variance inflation factor.

### 3.4. Structural Equation Modeling

**Figure 3:**
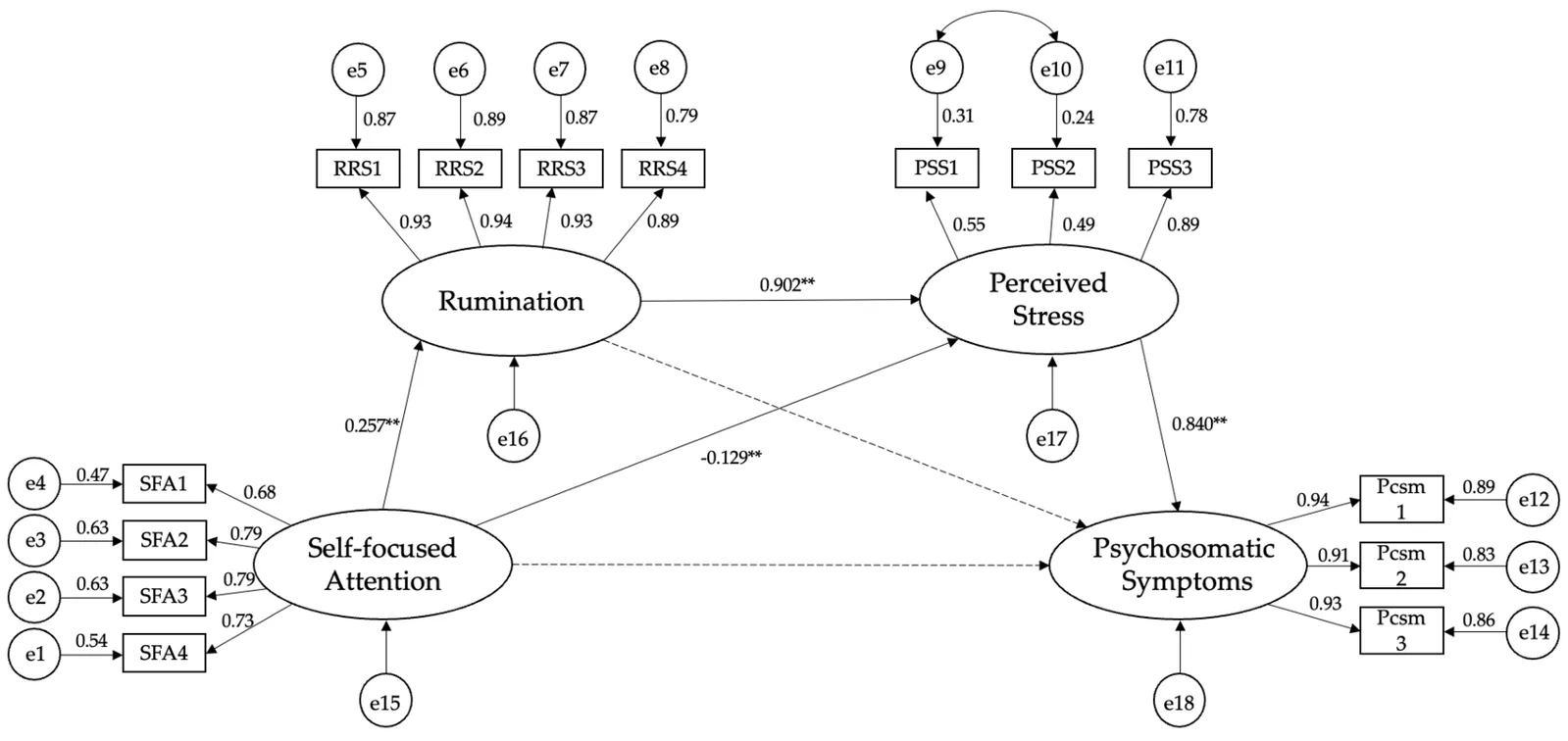
The Structural Equation Model Regarding the Mediating Effects of Rumination and Perceived Stress in the Relationship of SFA to Psychosomatic symptoms. Note. ** *p* < 0.001.

The hypothesized mediation model was tested using Structural Equation Modeling (SEM). To account for shared wording variance between two indicators of the same construct, the measurement errors were allowed to covary (i.e., residual covariance between PSS1 and PSS2), a theoretically defensible adjustment suggested by the modification indices; this yielded a small improvement in absolute fit while leaving the pattern of structural paths unchanged. The fit indices of the final model were as follows: χ^2^ [70] = 441.460, χ^2^/df = 6.307, CFI = 0.965, IFI = 0.965, TLI = 0.954, RMSEA = 0.080 [0.073–0.087], SRMR = 0.079, RMR = 0.037, as shown in Figure 3. Although the χ^2^/df value was relatively elevated and the RMSEA value was at the conventional threshold, model fit was evaluated cautiously based on the overall pattern of multiple indices, given the sensitivity of the chi-square statistic to sample size (Sathyanarayana & Mohanasundaram, 2024).

Some of the paths were significant. Specifically, SFA was positively related to rumination (β = 0.257, *p* < 0.001), and rumination was also positively related to perceived stress (β = 0.902, *p* < 0.001). Perceived stress was, in turn, significantly inversely related to SFA (β = −0.129, *p* < 0.001), and positively related to psychosomatic symptoms (β = 0.840, *p* < 0.001). The direct path between SFA and psychosomatic symptoms was not significant (β = 0.011, *p* = 0.781), suggesting that the association between SFA and psychosomatic symptoms may be predominantly indirect in the final model. In addition, the path between rumination and psychosomatic symptoms was not significant (β = 0.062, *p* = 0.619).

The mediating effect and the associated 95% confidence intervals are presented in Table 4. According to the results, the indirect association between SFA and psychosomatic symptoms was decomposed into three indirect paths. The specific indirect effect via rumination alone was not significant. By contrast, the indirect path via perceived stress and the chained indirect path through rumination and perceived stress were significant but showed opposite directions. Specifically, the indirect effect via perceived stress alone was negative, whereas the chained indirect effect was positive. This pattern suggests a suppression pattern (MacKinnon et al., 2000). These opposite directions indicate that perceived stress should not be interpreted as a simple independent pathway from SFA to psychosomatic symptoms; rather, its role should be understood in relation to rumination. The direct effect of SFA on psychosomatic symptoms was not significant, suggesting that the relationship was primarily indirect. To further clarify this pattern, supplementary PROCESS mediation analyses were conducted using scale composite scores, and detailed results are reported in Supplementary Table S4. When rumination was tested as the single mediator, the indirect effect of SFA on psychosomatic symptoms through rumination was positive and significant, whereas the remaining direct effect became negative, further supporting a suppression pattern. In contrast, perceived stress did not significantly mediate the association between SFA and psychosomatic symptoms when tested as the single mediator. These supplementary findings suggest that perceived stress did not act as an independent mediator but was mainly involved in the chained pathway with rumination.

A theoretically plausible reversed-order model, in which perceived stress preceded rumination, yielded highly similar fit indices to the hypothesized model. Detailed comparison results are presented in Supplementary Table S5.

**Table 4:** Bootstrapping Indirect effects and 95% confidence intervals (CI) for the meditational model.

Pathways	Estimate	SE	Percentage	95% CI	*p*
SFA-Rumination-Psychosomatic symptoms	0.016	0.038	14.2%	[−0.066, 0.072]	0.588
SFA-Perceived stress-Psychosomatic symptoms	−0.108	0.038	−95.8%	[−0.193, −0.048]	0.001
SFA-Rumination-Perceived stress-Psychosomatic symptoms	0.194	0.051	171.9%	[0.116, 0.310]	0.000
Total indirect effect	0.102	0.051	90.3%	[0.001, 0.195]	0.049
Direct effect	0.011	0.035	9.7%	[−0.049, 0.086]	0.781
Total effect	0.113	0.044	100%	[0.023, 0.197]	0.016

## 4. Discussion

The present study identified SFA, rumination, and perceived stress as key psychological factors associated with psychosomatic symptoms. Although current interventions, such as compassion-focused therapy (CFT) and gratitude interventions, are effective in alleviating symptoms such as anxiety and depression, they tend to be broad in scope and fail to clearly specify the specific psychological constructs they target (Boggiss et al., 2020; Gilbert, 2009). Research has demonstrated the positive effects of positive psychology interventions (PPIs), which not only effectively enhance well-being in clinical samples with psychosomatic disorders but can also treat common psychological symptoms such as depression and anxiety (Chakhssi et al., 2018). However, this lack of specificity regarding target variables limits the ability to guide the design of more targeted interventions.

There was a significant positive association between SFA and rumination. This finding was consistent with the Response Styles Theory (Nolen-Hoeksema, 2003). This theory posits that in response to negative affects, individuals persistently focus their attention on their own feelings, problems, and their consequences. In other words, SFA provides the attentional resources and cognitive focus required for rumination. Furthermore, this positive association aligned with the Self-Regulatory Executive Function (S-REF) model (Matthews & Wells, 2003), which regards self-focus as a component of a cognitive syndrome that promotes repetitive self-referential processing. However, rumination demonstrated no significant mediating effect between SFA and psychosomatic symptoms, which contradicted the hypothesis H1 of this study. On the one hand, this finding aligned with some existing research on the Symptom Perception Hypothesis (Ongaro & Kaptchuk, 2019; Van Wijk & Kolk, 1997). Given that an individual’s symptom perception operates by regulating their attention and interpretation of ambiguous bodily signals. Symptoms may not be perceived in the absence of certain subjective perceptual processes. On the other hand, this phenomenon might be related to the extremely strong association between perceived stress and psychosomatic symptoms in the model. Specifically, this study found that both the independent mediation effect of perceived stress and the chained mediation effect involving rumination and perceived stress were highly significant in the relationship between SFA and psychosomatic symptoms.

This study found that perceived stress mediated the relationship between SFA and psychosomatic symptoms, a finding consistent with the Control Theory of Self-Regulation and the Reflective Self-Focus theory (Carver & Scheier, 1981; Trapnell & Campbell, 1999). As a core mechanism of self-regulation, SFA is primarily used to detect the gap between an individual’s current state and their target standards. In the absence of rumination, that means not stuck in a repetitive, negative state of gap fixation. Such gap detection will trigger effective goal-directed behaviors, such as strategy adjustments or resource seeking, thereby enhancing an individual’s sense of control over the environment and eventually reducing their subjective assessment of stress. Concurrently, the dual distinction of SFA indicated that, without rumination, SFA primarily operated as reflective self-focus, which is a non-judgmental form of self-exploration rooted in cognitive curiosity. It maintained a stable negative association with perceived stress and reduced psychosomatic symptoms. Existing research also supports that healthy self-focus, such as mindfulness observation, is significantly associated with perceived stress (Creswell, 2017).

In terms of the chain-mediated pathway, rumination further intensified psychosomatic symptoms by strengthening perceived stress. This chain pathway was consistent with the perseverative cognition hypothesis (PCH), which states that prolonged cognitive engagement with stressors, such as rumination, will lead to sustained physiological activation and ultimately adverse health outcomes (Brosschot et al., 2006). This hypothesis emphasizes how enduring cognitive processes extend the stress experience, affecting both psychological and physical aspects (Ottaviani et al., 2016). According to cognitive appraisal theory, rather, the stressor itself does not determine an individual’s response, instead, how the individual evaluates it is the determining factor (Lazarus & Folkman, 1984). After engaging in ruminative thought patterns, cortisol reactivity to stress increases (Hilt et al., 2015), which in turn indirectly influences psychosomatic symptoms. This interpretation was also consistent with prior research showing that rumination is closely associated with heightened stress perception and prolonged stress responses (Gianferante et al., 2014). Researchers have also noted that the physiological responses occurring during the stressor may be less significant than the cognitive, representational rumination that may occur long after the stressor itself has ended (Brosschot et al., 2006), further supporting the central role of perceived stress in predicting psychosomatic health outcomes.

In summary, the relationship between SFA and psychosomatic symptoms was consistent with a full mediation pattern through rumination and perceived stress. The effect of SFA on psychosomatic symptoms tended to arise from changes at the level of psychological processing. That is, SFA itself was not directly associated with psychosomatic symptoms in the final SEM; rather, its association with symptoms appeared to depend on psychological processes such as rumination and the perception of stress, instead of a direct symptom-inducing effect of SFA itself. This model suggested that SFA is a process of directing attention toward internal stimuli during social interactions, and its association with psychosomatic symptoms is more likely to depend on post-event processing rather than a direct relationship (Gaydukevych & Kocovski, 2012). After including mediators, the direct effect of SFA was weakened and even showed non-significance, suggesting that the association between SFA and psychosomatic symptoms may be better explained by related psychological processes than by SFA alone. Furthermore, the suppression effect observed in the model suggested that the association between SFA and psychosomatic symptoms should not be understood as a single linear pathway. Rather, SFA appeared to have different implications depending on whether it was linked to rumination. In this sense, rumination may provide the cognitive context in which SFA becomes connected to heightened stress appraisal, such that perceived stress is better understood not as a simple bridge from SFA to psychosomatic symptoms but as a stress-appraisal process that becomes particularly relevant when SFA is accompanied by rumination, thereby linking these processes to psychosomatic symptoms (Hilt et al., 2015; Nolen-Hoeksema et al., 2008).

Although the hypothesized sequence from rumination to perceived stress was retained because it is consistent with the perseverative cognition hypothesis, the reversed sequential model showed comparable fit. PCH suggests that repetitive negative thinking, including rumination, may prolong stress-related cognitive, affective, and physiological activation (Brosschot et al., 2006). Consistent with this view, post-stress rumination has been found to predict prolonged HPA-axis responses to repeated acute stress (Gianferante et al., 2014), and rumination has been described as a maladaptive response style that can intensify negative thinking and emotional distress (Nolen-Hoeksema et al., 2008). At the same time, perceived stress and rumination are closely related and may reinforce one another, as higher perceived stress has also been associated with greater rumination (Willis & Burnett, 2016). Therefore, the present findings should not be interpreted as definitive evidence of a fixed temporal sequence. Rather, viewing rumination and perceived stress together may provide a more integrated understanding of how self-focused attention is associated with psychosomatic symptom burden. Future longitudinal or experimental studies are needed to examine whether these processes operate sequentially, reciprocally, or both.

These findings held particular significance for female health, as they highlighted that beyond physiological vulnerabilities, female psychosomatic symptoms may also be associated with identifiable cognitive processes. From this perspective, the observed pattern of associations offered meaningful insights into the psychological factors underlying female psychosomatic health issues. This extended existing research by highlighting a more specific pattern of associations among SFA, rumination, perceived stress, and psychosomatic symptoms.

### 4.1. Limitations

Although this study offers valuable insights, several limitations should be acknowledged. First, the cross-sectional design makes it difficult to establish temporal sequence or causality among variables. Psychosomatic symptoms may be associated with cognitive processes, including higher levels of SFA, rumination, or perceived stress, which suggests potential bidirectional effects. Although the results indicate patterns of mediation that are consistent with our theoretical model, causal direction cannot be established. A clearer understanding of this complex framework will require future longitudinal or experimental designs. In addition, a cross-sectional study captures only participants’ psychological states at a specific point in time. The statistics may reflect transient emotions or temporary stressors that may not fully represent stable and enduring cognitive traits. As all variables were measured using self-report instruments, future studies may benefit from incorporating data collected at multiple time points or using complementary assessment approaches to further strengthen the robustness of the findings. Furthermore, although the sample included women from a range of occupational and demographic backgrounds, the use of online convenience sampling may still have introduced self-selection bias. In addition, the relatively limited age distribution of the sample may constrain the extent to which the findings can be generalized across different stages of adulthood. Therefore, careful consideration should be exercised when generalizing the findings of this study to a broader population of women. Future research should employ more diverse, representative, or stratified sampling methods to increase sample heterogeneity and strengthen demographic representativeness, which may help to explore possible developmental differences across age groups. Although the final SEM showed generally favorable incremental and residual fit indices in the present sample, the relatively high χ^2^/df value and borderline RMSEA suggest that the model should be interpreted cautiously. In particular, future research may consider incorporating broader socioeconomic and health-related variables to further examine these relationships. Finally, all participants shared the same cultural background, so replicative studies across diverse sociocultural contexts are necessary to test whether the observed association patterns exhibit cross-cultural universality.

### 4.2. Clinical and Practical Implications

The findings of this study offer valuable insights into possible directions for future intervention research on female psychosomatic symptoms. However, given the cross-sectional nature of the present study, these implications should be considered preliminary and hypothesis-generating rather than definitive clinical recommendations. Specifically, the present findings suggest that SFA was not directly associated with psychosomatic symptoms in the final SEM. Instead, its association with psychosomatic symptoms appeared to be primarily indirect and related to rumination and perceived stress. Therefore, future intervention research may consider whether focusing on ruminative processes, rather than simply reducing self-focused attention, is a useful approach for addressing psychosomatic symptom burden. Psychoeducation may also explore ways to help individuals distinguish between reflective self-focus and repetitive, critical ruminative self-focus, although the adaptive value of different forms of self-focus should be examined more directly in future studies. Second, although perceived stress is positively correlated with psychosomatic symptoms, the mediation findings suggest that its role should be interpreted in relation to rumination rather than as a simple independent pathway. Therefore, future intervention studies may examine whether helping individuals appraise stress more flexibly and reduce rumination-related stress amplification is beneficial for psychosomatic symptom management. Third, the significant chain-mediated effect points to the potential value of examining multi-target approaches in future research, such as reducing rumination, reappraising perceived stress, and establishing goal-oriented coping strategies. However, experimental or longitudinal studies are needed before these approaches can be considered evidence-based intervention components. Finally, in preventive interventions, the combination of high rumination and high perceived stress may be worth examining as a potential risk profile for psychosomatic symptoms, rather than focusing solely on rumination. This may help inform future low-intensity early intervention studies.

From an assessment perspective, our findings may support more comprehensive approaches to the assessment of female psychosomatic health. More broadly, the present model may help generate hypotheses for future work seeking to identify which cognitive and stress-related processes are most relevant to psychosomatic symptoms in females.

## 5. Conclusions

In summary, this study suggests that SFA is not necessarily detrimental in relation to psychosomatic symptoms. Rather, its association with psychosomatic symptoms appears to be primarily indirect and linked to cognitive processing. Specifically, the non-significant direct path and the significant chained indirect path suggest that SFA was associated with psychosomatic symptoms mainly in the context of rumination and perceived stress. The negative indirect path through perceived stress alone should be understood as part of the suppression pattern, indicating that perceived stress did not operate as a straightforward independent pathway from SFA to psychosomatic symptoms in this model. These findings highlight that rumination and perceived stress are important psychological processes involved in the association between SFA and psychosomatic symptoms. Furthermore, the results suggest the potential relevance of interventions targeting rumination and perceived stress, although longitudinal and experimental studies are needed to determine whether modifying these processes can reduce psychosomatic symptoms, particularly among females.

## Figures and Tables

**Table 1:** Demographic characteristics of participants (n = 839).

Variables	Category	n	Percentage (%)
Age	M (SD)	26.50 (6.08)	
	Range	18–59	
Marital status	Single	564	67.2
	Married	270	32.2
	Divorced	5	0.6
Occupational status	Student	218	26.0
	Freelance worker	57	6.8
	Administrative staff	57	6.8
	Product/Operations staff	50	6.0
	Teacher	44	5.2
	self-employed individual	43	5.1
	Other (each < 5%, including marketing/sales, finance, engineering, service workers, manual labor, agricultural workers, healthcare professionals, and others)	370	44.1

Note. M Mean, SD Standard Deviation.

## Data Availability

The datasets used and/or analyzed in this study may be made available upon reasonable request to the corresponding author.

## Supplementary Materials

The following supporting information can be downloaded at: https://www.mdpi.com/article/10.3390/bs16081266/s1, Table S1: Item-level CFA Results Based on Original Scale Structures of SFAS, RRS-CV, and PSS-10; Table S2: Parcel Composition and Item Assignment Matrix; Table S3: Exploratory Factor Analysis of GAD-7 and PHQ-15 Items; Table S4: Single-mediator PROCESS Analyses; Table S5: Comparison of the Hypothesized and Alternative Mediation Models.

## Abbreviations

The following abbreviations are used in this manuscript:

M	Mean
SD	Standard Deviation
SEM	Structural Equation Modeling
HPA axis	Hypothalamic–pituitary–adrenal axis
ACTH	Adrenocorticotropic hormone
SFA	Self-focused Attention
PMDD	Premenstrual Dysphoric Disorder
SFAS	Self-Focused Attention Scale
RRS-CV	Chinese version of the Ruminative Response Scale
PSS-10	Perceived Stress Scale
GAD-7	Generalized Anxiety Disorder-7
PHQ-15	Patient Health Questionnaire-15
CMB CFA	Common Method Bias Confirmatory Factor Analysis
VIF	Variance Inflation Factor
CFT	Compassion-focused Therapy
PPI	Positive Psychology Interventions
PCH	Perseverative Cognition Hypothesis
S-REF	Self-Regulatory Executive Function
